# Unambiguous Identification of Glucose-Induced Glycation in mAbs and other Proteins by NMR Spectroscopy

**DOI:** 10.1007/s11095-022-03454-0

**Published:** 2022-12-13

**Authors:** Jennifer E. Moises, Christof Regl, Arthur Hinterholzer, Christian G. Huber, Mario Schubert

**Affiliations:** 1 Department of Biosciences and Medical Biology, University of Salzburg, Hellbrunner Strasse 34, 5020 Salzburg, Austria; 2 Christian Doppler Laboratory for Innovative Tools for Biosimilar Characterization, University of Salzburg, Hellbrunner Strasse 34, 5020 Salzburg, Austria

**Keywords:** Amadori product, Glycation, NMR spectroscopy, Mass spectrometry, Monoclonal antibody, NMR spectroscopy, Post-translational modifications

## Abstract

**Objective:**

Glycation is a non-enzymatic and spontaneous post-translational modification (PTM) generated by the reaction between reducing sugars and primary amine groups within proteins. Because glycation can alter the properties of proteins, it is a critical quality attribute of therapeutic monoclonal antibodies (mAbs) and should therefore be carefully monitored. The most abundant product of glycation is formed by glucose and lysine side chains resulting in fructoselysine after Amadori rearrangement. In proteomics, which routinely uses a combination of chromatography and mass spectrometry to analyze PTMs, there is no straight-forward way to distinguish between glycation products of a reducing monosaccharide and an additional hexose within a glycan, since both lead to a mass difference of 162 Da.

**Methods:**

To verify that the observed mass change is indeed a glycation product, we developed an approach based on 2D NMR spectroscopy spectroscopy and full-length protein samples denatured using high concentrations of deuterated urea.

**Results:**

The dominating β-pyranose form of the Amadori product shows a characteristic chemical shift correlation pattern in 1H-13C HSQC spectra suited to identify glucose-induced glycation. The same pattern was observed in spectra of a variety of artificially glycated proteins, including two mAbs, as well as natural proteins.

**Conclusion:**

Based on this unique correlation pattern, 2D NMR spectroscopy can be used to unambiguously identify glucose-induced glycation in any protein of interest. We provide a robust method that is orthogonal to MS-based methods and can also be used for cross-validation.

**Supplementary Information:**

The online version contains supplementary material available at 10.1007/s11095-022-03454-0.

## Introduction

Spontaneous post-translational modifications (PTM) that occur during expression or storage of therapeutic proteins can alter the function, efficacy, life-time and might lead to side effects. After the first therapeutic monoclonal antibody (mAb) was approved in 1986 [1], these therapeutics became increasingly important over the years. Nowadays, therapeutic mAbs are indispensable in medicine and offer many new therapies, especially for hard-to-cure or, so far, incurable diseases, such as certain types of cancer [2] (https://www.antibodysociety.org/resources/approved-antibodies/). Therapeutic mAbs are typically post-translationally modified and, as for any other drugs, the producers and the authorities have to monitor quality and consistency closely.

Changes/variations of the therapeutic proteins that are critical for safety and efficacy, so called critical quality attributes (CQA)[3], are of particular interest. Quite often these are related to PTMs, such as glycosylation, glycation, oxidation or deamidation. Understanding the mechanism of their formation, and finding methods for their detection and quantification are crucial for keeping their abundances within defined tolerances and thus ensuring the safety and efficacy of a therapeutic protein.

PTMs are typically investigated by high performance liquid chromatography (HPLC) coupled to mass spectrometry (MS) either with bottom-up, top-down or middle-up approaches [4–6]. However, distinguishing two isobaric PTMs is often challenging due to the same mass. Therefore, complementary methods to identify and potentially quantify these PTMs are required.

An alternative approach for the detection of different PTMs is based on 2D NMR spectroscopy under denaturing conditions [7–12]. Two recent developments enabled to overcome limitations of the large size of mAbs and the associated severe line broadening. ^1^H-^13^C HSQC spectra are either used to detect methyl groups, which display sharper line widths due to favorable relaxation properties caused by fast methyl group rotation [13–16], or samples are measured under denaturing conditions using high concentrations of urea [17]. The methyl-HSQC spectra serve as fingerprint spectra, which are ideally suited to compare different batches of mAbs. However, the detected methyl groups will only sense PTMs indirectly. The second approach, which includes denaturing the protein with 7 M deuterated urea in D_2_O, is based on line-narrowing due to the significantly higher flexibility of denatured protein chains that is especially suited to detect PTMs. Under denaturing conditions, the ^1^H-^13^C HSQC spectra are simplified dramatically resulting only in random coil chemical shift correlations of the 20 proteinogenic amino acids that are well known [18]. Any modification results either in additional signals, or lead to significant alterations of the amino acid signals to which the PTM is attached. Ideally, each PTM leads to a characteristic chemical shift correlation pattern that is distinguishable from random coil correlations of the proteinogenic amino acids within a protein. PTMs with a characterized NMR signature include pyroglutamate formation [9], oxidation [8], deamidation of Asn and strand cleavage after Asp [7, 10], terminal ends of glycans including high-mannose and certain types of complex glycans [11, 12, 17, 19] and the immunogenic α-Gal epitope [20].

**Fig. 1:**
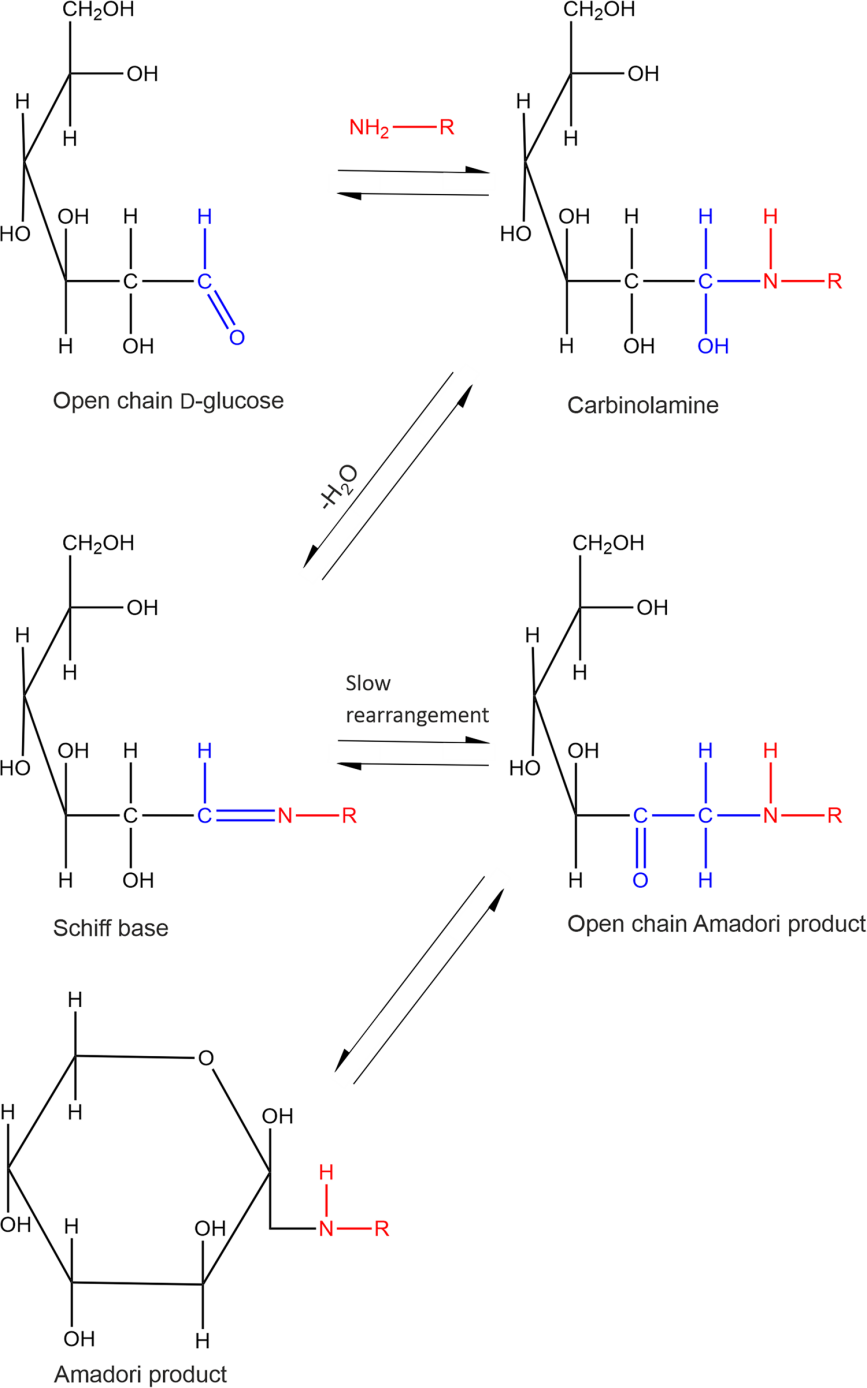
Mechanism of glycation illustrated by the reaction of glucose with a primary amine.

**Fig. 2:**
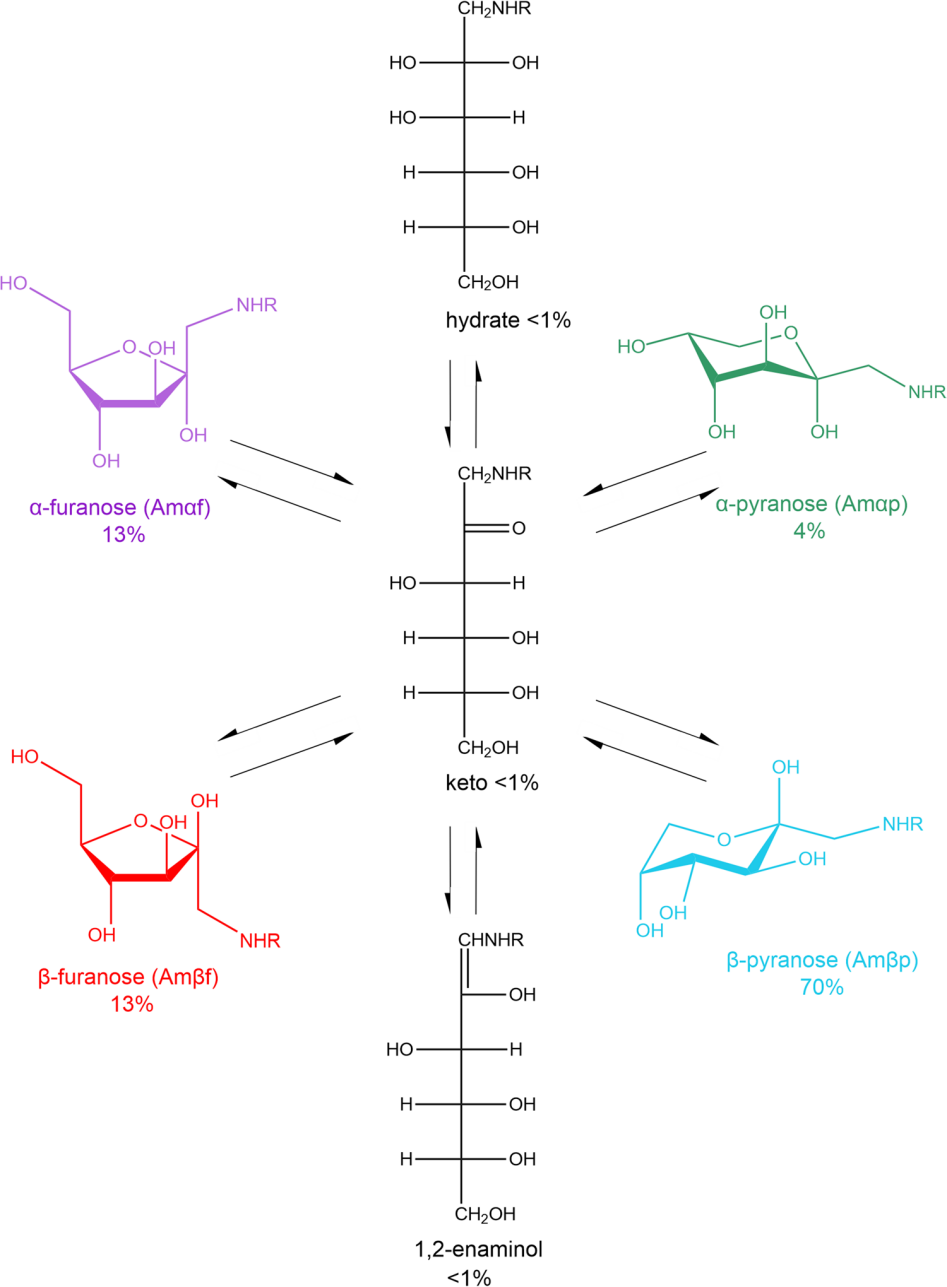
The glycation product fructoselysine exists in an equilibrium of different forms. The β-pyranose form is the most dominant one with an abundance of about 70%. The two furanose forms have an abundance of 13% and the β-pyranose form only 4%. The open, acyclic forms are very rare (less than 1%).

Glycation products are non-enzymatic PTMs originating from the spontaneous reaction between a reducing sugar, such as glucose, with free amine groups, which are found abundantly in lysine side chains (Fig. 1). This stands in stark contrast to the abundant N- and O-glycosylation that are under enzymatic control and tightly regulated in cells [21, 22]. Glycation *in vivo* is typically associated with aging, diabetes, atherosclerosis, neurodegenerative diseases and chronic renal failure [23]. Glycation itself is the first step of the so called Maillard reaction [24]. The glycation mechanism involves the nucleophilic attack of a free primary amine at the carbonyl group of a reducing sugar resulting in a Schiff base. Due to the Amadori rearrangement, the product formed from glucose and a lysine side chain slowly converts to the Amadori product fructoselysine, which exists in equilibrium between different forms (Fig. 2). The most stable forms are cyclized versions of the Amadori product, two pyranose and two furanose forms [24, 25]. In the case of Nϵ-fructose-Nα-formyl-lysine, the β-pyranose form is dominant with an abundance of ~ 70%, followed by the α- and β-furanose forms with both ~ 13% abundance [26]. The α-pyranose form is only sparsely populated with ~ 4% and the open forms are very rare (< 1%). The Nϵ-fructose-Nα-formyl-lysine model system is structurally closest to a protein context. The Amadori products between glucose and the α-amino groups of isolated amino acids show similar populations of the different Amadori product forms [27]. However, the equilibrium has not been investigated in the context of a real protein so far, in which several groups can react with glucose.

While the Amadori products are reported to be reversible [28], the subsequent advanced glycation end products (AGEs) are not [29]. These AGEs can be more harmful than the rather benign Amadori products. Since glycation is reversible, the amount of glycation products depends on the concentration of reactive sugars, as well as the reactivity of the glycated moiety [30]. Therapeutic mAbs are typically expressed using media that contain such reactive sugars like glucose. Lysine Nε groups are the most glycated entities in proteins and the lower their pKa value, the higher is their reactivity towards reducing sugars [28]. Thus the pKa decides about the amount of glycation in a protein in equilibrium at a certain glucose concentration. For example, a mAb containing a Lys with the unusually low pKa of 6.7 reaches a steady state glycation level of approx. 24% after 14 days in the blood stream of a non-human primate with a blood glucose level around 5.6 mM [28]. This glycation level agrees with the prediction based on the pKa and the glucose concentration. Interestingly, the level of glycation is in a similar range as for human serum albumin [31], suggesting similarities. The kinetics of glycation and de-glycation are rather slow. The process of glycation takes a few days at a high glucose concentration at 310 K and a pH of 7 to reach equilibrium [28]. Deglycation, however, can take from 10 to more than 40 days, depending on the temperature, the pH and the concentration of sugar. The higher the pH and the temperature, the faster de-glycation is. Neighboring amino acids also strongly influence the susceptibility for glycation, as hydrophobic or acidic amino acids increase the reactivity of lysine, likely due to their influence on the pKa and the accessibility [32, 33]. There is no clear protein sequence preference for glycation, but there seems to be a slight preference for certain amino acid types around the glycation site [34, 35].

Because glycation is considered a critical quality attribute (CQA) in therapeutic mAbs, especially in the antigen-binding region of the Fab part [36, 37], it is important to monitor if this modification is present and if it stays within a certain tolerance range. The mass difference for glycation is isobaric to an additional hexose attached to a glycan [38]. For very complicated glycosylation patterns, it is especially challenging to distinguish between glycation and an additional hexose attached to a glycan.

The aim of this study was to investigate glycation products in the context of proteins in order to elucidate characteristic chemical shift patterns that can later be used for a fast and unambiguous identification of glycation in therapeutic proteins by 2D NMR spectroscopy. Here, we developed an NMR approach to distinguish glycation from glycosylation variants. This approach is based on 2D ^1^H-^13^C correlation spectra. Characteristic signals of the major Amadori product were identified, which are suited to unambiguously identify the presence of glycation with glucose. As this approach is complementary to MS-based methods, it is suited as a gold-standard for cross-validation.

## Material and Methods

### General Procedure for the Glycation of Proteins

Protein samples were dissolved in 10 mM KH_2_PO_4_/K_2_HPO_4_ pH 6.0, 5 mM NaCl buffer and 0.5 M glucose solution and incubated for at least 5 days at 40°C. After incubation bovine serum albumin (BSA, Sigma A7030) and lysozyme (Fluka 62,971) were dialyzed against ddH_2_O overnight in a SpectraPor 3.5 kDa cutoff membrane. Since aprotinin was too small for the membrane cutoff, we used an ultrafiltration device with a cutoff of 1 kDa (Pall Microsep advance FE6846/MCP001C41). All samples where lyophilized and then dissolved in 500 µL of a 7 M urea-d_4_ solution in D_2_O. The pH* (unadjusted pH reading in D_2_O) was adjusted to 7.4 by adding 3% DCl in D_2_O (Armar Chemicals 042,100.0035). To reduce the disulfide bonds DTT-d_10_ (Cambridge Isotope Laboratories) was added to a concentration that exceeded the concentration of contained cysteine by a factor of 1.5 to 2. The sample was heated to 60 °C for 15 min.

### Individual Procedures for Glycation

A volume of 6 ml of a rituximab formulation with a concentration of 10 mg/mL (MabThera®, Hoffmann-La Roche, N7025B04 exp. 2/2017; H0102B06 exp. 05/2014) was mixed with 0.72 g glucose dissolved in 2 mL H_2_O. For adalimumab (Humira®, AbbVie; exp. 2016), 2 mL of formulation buffer containing 10 mg/mL was mixed with 8 mL of a 0.625 M solution of glucose. The samples were incubated at 40°C for 7 days. Dialysis, lyophilization and dissolving under denaturing conditions followed the general procedure resulting in a final concentration of 30 mg/mL corresponding to a concentration of 0.2 mmol/L. The disulfide bridges were reduced by adding tris(2-carboxyethyl)phosphine hydrochloride (TCEP; Sigma Aldrich) and heating to 60°C for 15 min.

### Sample Preparation of Untreated Proteins

A volume of 4 mL of cetuximab formulation (Erbitux®, Merck KGaA, Lot. 208,480, exp. 09/2019) was dialyzed against ddH_2_O using a SpectraPor membrane with a cut-off of 3.5 kDa. After lyophilization overnight, the sample was dissolved in 500 µL of 7 M urea-d_4_ solution in D_2_O resulting in a final concentration of 40 mg/mL mAb. The disulfide bridges were reduced by adding tris(2-carboxyethyl)phosphine hydrochloride (TCEP; Sigma Aldrich) and heating to 60°C for 15 min.

For denosumab (Prolia Amgen; Batch Nr.: 1,067,099, Exp Date.: 11/18), the formulation corresponding to 20 mg was exchanged to ddH_2_O by an ultrafiltration device (Amicon, cut off 10 kDa). After that the sample was lyophilized overnight and then dissolved in 7 M deuterated urea-d_4_, the disulfide bridges were reduced with TCEP. The final concentration of the sample was 36 mg/mL.

### NMR

Unless stated otherwise, spectra were recorded using a 600 MHz Bruker Avance III HD spectrometer equipped with a ^1^H/^13^C/^15^N/^31^P quadruple-resonance room temperature probe at 298 K. Some spectra were measured either on a 500 MHz Bruker Avance III HD spectrometer equipped with a TCI ^1^H/^13^C/^15^N triple-resonance cryo-probe, 700 MHz Bruker Avance III HD spectrometer equipped with a QCI ^1^H/^13^C/^15^N quadruple-resonance cryo-probe, or a 900 MHz Bruker Avance spectrometer equipped with a TCI ^1^H/^13^C/^15^N triple-resonance cryo-probe. All samples were measured in a standard 5 mm NMR tube (Armar, Type 5TA) with a volume of 500 µL.

For assigning the resonances of the Amadori products, the following 2D experiments were recorded: ^1^H-^13^C HSQC, ^1^H-^13^C HMBC (hmbcgpndqf), ^1^H-^1^H TOCSY with mixing times of 100 ms and 12 ms, ^1^H-^1^H COSY (cosygpppqf), ^1^H-^13^C HSQC-TOCSY (hsqcdietgpsisp.2) with mixing times of 13 ms and 100 ms. More details of the experimental parameters are given in the Figure captions. The data was processed with Topspin 3.6.2 (Bruker) and analyzed with Sparky 1.470 [39]. Spectra of human serum albumin and bromelain were measured as reported previously [17].

### Quantification

For estimating the quantity of glycation, we compared integrals of representative signals in the ^1^H-^13^C HSQC spectra. Isolated signals were integrated using a Gaussian fit in Sparky (D. Goddard and D. G. Kneller, “SPARKY 3,” University of California, San Francisco, 2000.). The volumes of isolated ^1^H-^13^C correlations of the β-pyranose form were compared to isolated and well-integrable signals of the denatured protein. The latter could be either a typical random-coil signal resulting of a known amount of residues of an amino acid type in the protein sequence, e.g. a Cγ-Hγ correlation of Gln, or the rare case of an isolated signal of a single amino acid. Such isolated signals can occur due to the strong influence of a proline on the chemical shifts of its preceding residue [18]. The volume corresponding to a single proton – a normalized volume – was obtained by dividing the integral by the occurrence of the particular amino acid type in the sequence and, if necessary, by the factor of 2, in case a CH_2_ group resulted in one signal. Because a standard ^1^H-^13^C HSQC experiment is set up in a way to give approximately the same intensities for CH, CH_2_ and CH_3_ groups by using an INEPT delay of 3.4 ms, which is a compromise for all multiplicities, signal integrals between different multiplicities are not comparable and thus the signals are not strictly quantitative. However, comparison of integrals among the same multiplicity is nearly quantitative assuming similar ^1^*J*_CH_ coupling constants, a similar T_1_ relaxation rate, and sufficiently long recycle delays. Therefore, signal volumes of CH groups of the glycation product were only compared to volumes of CH signals of the protein. Signal volumes of the CH_2_ group of fructolysine were only compared to volumes of CH_2_ groups of the protein.

In the case of glycated BSA, the isolated Cα-Hα signal of Ser at the unique position 56.6 ppm/ 4.71 ppm (preceding proline) was used as a reference integral for CH signals, and the Cδ-Hδ signal of all 23 arginines was used as reference for CH_2_ groups. In the case of aprotinin, the two sets of C-H signals corresponding to slightly different β-pyranose forms were compared to the volume of the Cα-Hα signal of four phenylalanines in the sequence. The CH_2_ signals of fructolysine of glycated aprotinin were compared to the integral of Cγ-Hγ of the single glutamine in the sequence. For lysozyme, we decided to use the unique Cα-Hα signal of a threonine followed by a proline at the characteristic position of 60.1 ppm/ 4.60 ppm as reference for CH signals. The signals of the CH_2_ group of fructolysine were very weak in the applied multiplicity-edited HSQC and displayed a coupling pattern. They were therefore not well suited for integration. In the case of glycated rituximab, only the CH_2_ signals of fructolysine were isolated and could be integrated. We compared the volumes to the Cγ-Hγ signal of glutamic acid, which we divided by the number of glutamic acids in the sequence [31], and by 2 (CH_2_ with a single ^1^H resonance).

For the naturally glycated proteins HSA and Bromelain, we used the volume of the Cβ-Hβ signal of isoleucines (9 × in HSA and 20 × in bromelain) as reference for CH signals and the Cα-Hα signal of glycines (13 × in HSA and 28 × in bromelain) as reference for CH_2_ signals.

### Sample Preparation for Mass Spectrometry

Porcine aprotinin was dissolved at a concentration of 0.920 mmol^.^L^−1^ in a 10.0 mmol^.^L^−1^ KH_2_PO_4_ buffer at pH 6.0 with 5.0 mmol^.^L^−1^ NaCl and 500.0 mmol^.^L^−1^ d-glucose. The solution was incubated for 1 week at 40°C on a thermo-cycler, followed by rebuffering by a 1 kDa cut-off filter (Microse™ from Pall Corporation) to 50 mmol^.^L^−1^ triethylammonium bicarbonate at pH 4.50 to a concentration of 0.20 mmol^.^L^−1^. An aliquot of 140 µg was processed with 10 mmol^.^L^−1^ tris(2-carboxyethyl)phosphine at 55°C for 15 min in order to reduce disulfides, followed by alkylation of the obtained thiol groups by addition of a final concentration of 20 mmol^.^L^−1^ iodoacetamide and incubation at 22°C for 10 min in the dark. Next, the reduced and alkylated aprotinin sample was purified using C18 pipette tips (Pierce™ C18 Pipette Tips from Thermo Fisher Scientific) according to the manufacturer's protocol. The purified sample was dried at 40°C in a vacuum centrifuge. In a next step, the sample was specifically proteolyzed after asparagine and aspartate residues using legumain as a digestion protease (asparaginyl endopeptidase, provided by Elfriede Dall, University of Salzburg) [40]. Specifically, the dried sample was resuspended in 50 mmol^.^L^−1^ triethylammonium bicarbonate at pH 4.50 to a concentration of 1 µg^.^µL^−1^, subsequently, legumain was added at a 1:10 ratio (*w/w*), followed by incubation at 37°C for 5 h.

### High-Performance Liquid Chromatography-Mass Spectrometry Settings

Chromatographic separation of 250 ng aprotinin and aprotinin digest was carried out on a Thermo Scientific™ UltiMate™ 3000 RSLCnano System using an Acclaim™ PepMap™ 100 C18 reversed phase HPLC column (500 × 0.075 mm i.d., d_p_ 3 µm, Thermo Scientific™). As solvents a 0.10% aqueous formic acid solution (solvent A) and a 0.10% formic acid in acetonitrile solution (solvent B) were delivered at a flow rate of 200 nL^.^min^−1^. Intact aprotinin was separated by a linear gradient from 1.0–60.0% B in 10.0 min. The legumain digested aprotinin was separated by changing the solvents in the following manner: 1.0% B for 5.0 min, a linear gradient from 1.0–7.0% B in 5.0 min, a second linear gradient from 7.0–25.0% B in 30.0 min and a third from 25.0–45.0% B in 20.0 min. Both separation methods included flushing of the column at 80.0% B for 10 min and column re-equilibration at 1.0% B for 60 min. The column temperature was kept constant at 50°C. The nanoHPLC system was hyphenated to a Thermo Scientific™ Q Exactive™ Plus Hybrid Quadrupole-Orbitrap™ mass spectrometer via a Thermo Scientific™ Nanospray Flex™ ion source. The source was equipped with a SilicaTip emitter with 360 µm o.d., 20 µm i.d. and a tip i.d. of 10 µm (CoAnn Technologies Inc.). The spray voltage was set to + 1.5 kV, the S-lens RF level to 55.0 and the capillary temperature to 320°C. The intact aprotinin was analyzed in full scan mode at a scan range of *m/z* 500–4,000 and a resolution setting of 70,000 at *m/z* 200. The automatic gain control (AGC) target was set to 3e6 charges with a maximum injection time of 120 ms. For the digested aprotinin, each scan cycle consisted of a full scan at a scan range of *m/z* 350–2,000 and a resolution setting of 70,000 at *m/z* 200, followed by 15 data-dependent higher-energy collisional dissociation (HCD) scans using a 2.0 m*/z* isolation window for precursor isolation and 32% normalized collision energy for fragmentation with a resolution setting of 17,500 at *m/z* 200 for data acquisition. For the full scan, the AGC target was set to 3e6 charges with a maximum injection time of 100 ms, for the HCD scans the AGC target was 1e5 charges with a maximum injection time of 100 ms. Already fragmented precursor ions were excluded for 30 s. Data acquisition was conducted using Chromeleon™ 7.2 CDS (Thermo Scientific™), data evaluation was performed employing Thermo Scientific™ Xcalibur 4.2.28.14 and BioPharma Finder 3.0.62.11 (both Thermo Scientific™). Theoretical masses of aprotinin and fragments were calculated employing GPMAW 9.51 (Lighthouse data).

## Results

### Analysis of Artificially Glycated Model Proteins Under Denaturing Conditions

**Fig. 3:**
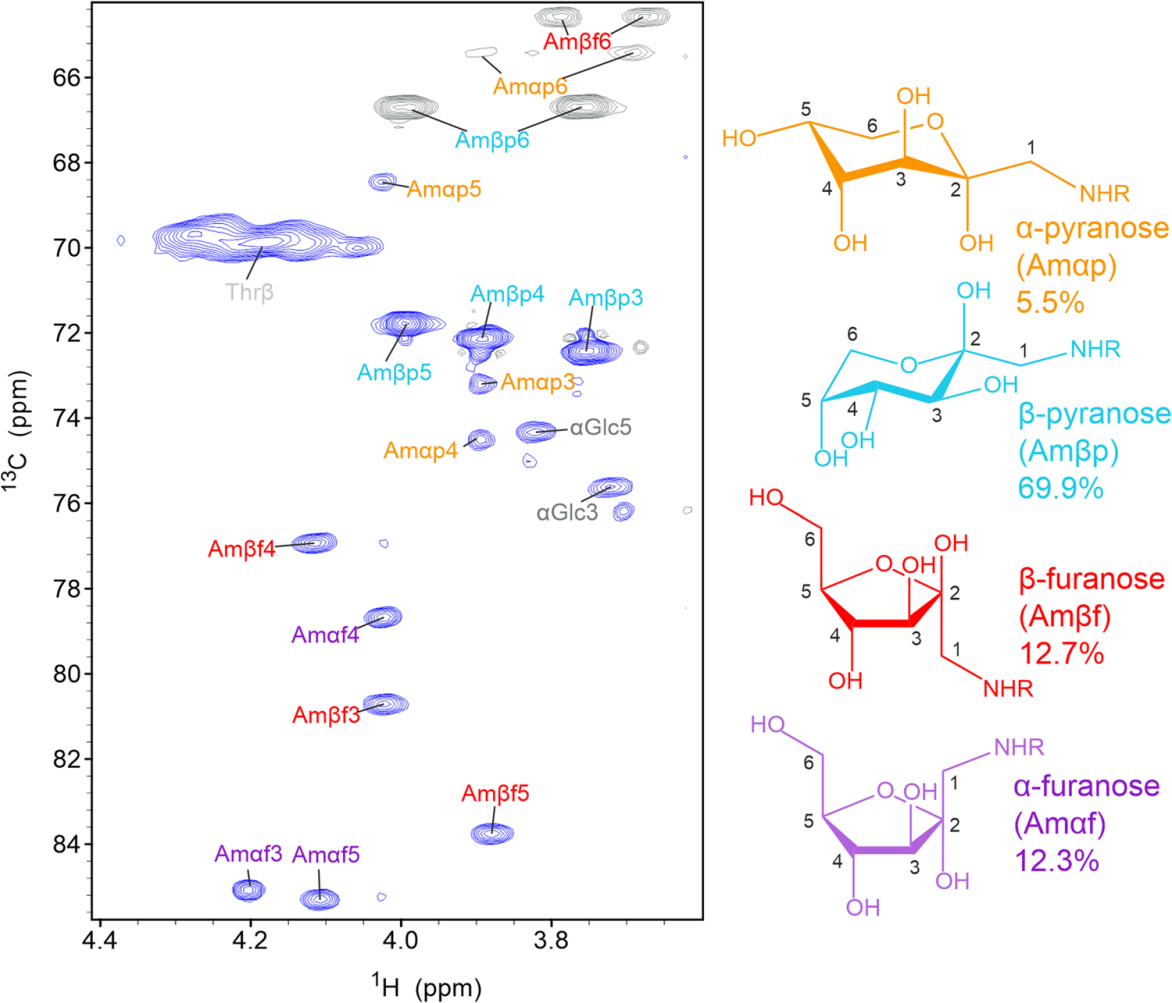
Glycation of the model protein BSA. ^1^H-^13^C HSQC spectrum of glycated bovine serum albumin (BSA) at a concentration of 2 mM measured with 128 transients, a recycle delay of 1 s and 2024 × 512 points. The sample was dialyzed against ddH_2_O, lyophilized and dissolved in 7 M urea-d_4_ in D_2_O pH* 7.4. The most dominant form of the Amadori product, the β-pyranose form is labeled in cyan. The furanose forms are labeled in red and purple. The chemical structures of these species are shown on the right using the same color code.

Although Amadori products were analyzed before using model compounds [26, 41], glycation of proteins has not been investigated by NMR spectroscopy so far. To obtain a sample with glycated lysine(s), we incubated protein and peptide solutions in the presence of phosphate with high concentrations of glucose at 40°C instead of using solid phase peptide synthesis using glycated precursors [41, 42]. Initial attempts with short peptides containing lysine and arginine gave only poor yields. However, bovine serum albumin (BSA), which was reported earlier to be prone for glycation with glucose [43], yielded a sample with large amounts of glycation products after incubation with 0.5 M glucose for 10 days. The main chain of BSA contains 59 Lysine residues, of which 16 were reported to be glycation sites [43]. After dialysis against ddH_2_O and lyophilization, the glycation was monitored by 2D NMR spectra measured under denaturing conditions using 7 M urea-d_4_ in D_2_O. In addition to the original protein signals and some remaining signals of free glucose, the ^1^H-^13^C HSQC spectrum showed several sets of signals that pointed to Amadori products (Fig. 3). The resonances of these species were assigned using a combination of ^1^H-^13^C HMBC, ^1^H-^1^H TOCSY, ^1^H-^1^H COSY, ^1^H-^13^C HSQC-TOCSY and a ^1^H-^13^C HSQC-COSY spectra resulting in the assignment of three species (Table I and Table S1). A comparison of the chemical shifts with previously reported data of the Amadori product of glucose and the amino acid lysine, namely Nϵ-(1-deoxy-d-fructos-1-yl)-Nα-formyl-l-lysine, also called fructoselysine [26], confirmed that the most abundant species is the β-pyranose form. The signals of the α- and β-furanose forms could be clearly assigned too, but their intensity is a factor 3–6 less intense in agreement to the previously reported abundances [26, 44]. Further comparison with assignments of Nα-(1-deoxy-d-fructos-1-yl)-l-alanine [27] and a peptide in which an N-terminal Lys was glycated at its side chain, [41] confirm these assignments to the different forms. There are small differences in the previously reported values, but this is likely due to the slightly different structures of the glycated moieties.

**Table I:** Experimental Chemical Shifts of the Most Abundant Form of the Amadori-Product, the β-pyranose Form, Observed in Glycated Model Proteins in Comparison to Previously Published Data (26, 27, 41, 45)

	BSA	Lysozyme	Aprotinin ^a^		Rituximab	Albumin human	Bromelain	Kaufmann 2016 ^b^	Kapczynska 2011^c^	Mossine 1994 ^d^	Mossine 2009 ^e^
C1^f^	(55.6)	n.d	n.d	n.d	(55.5)	55.5	55.5	54.5	55.6	55.5	48.0
C2	98.2	n.d	n.d	n.d	n.d	98.0	n.d	98.0	n.r	98.1	98.1
C3	72.4	72.5	72.4	71.8	72.4	72.4	72.5	72.6	72.2	72.3	72.4
C4	72.1	72.3	72.2	72.6	72.2	72.2	72.2	72.0	72.1	72.0	72.1
C5	71.8	71.9	71.8	72.0	71.8	71.8	71.8	71.6	71.5	71.6	71.7
C6	66.7	66.8	66.7	66.1	66.8	66.7	66.7	66.6	66.6	66.6	66.7
H1^f^	(3.29)	n.d	n.d	n.d	(3.27)	3.27	3.31	3.14	n.r	3.30	3.28
H1'	n.d	n.d	n.d	n.d	n.d	n.d	n.d	3.11	n.r	3.30	3.24
H3	3.75	3.78	3.78	3.78	3.75	3.75	3.79	3.57	n.r	3.73	3.75
H4	3.89	3.93	3.91	3.88	3.85	3.89	3.94	3.71	n.r	3.89	3.91
H5	4.00	4.03	4.01	3.97	3.99	3.99	4.03	3.82	n.r	4.01	4.03
H6	3.99	4.02	4.01	4.00	3.99	3.99	4.03	3.84	n.r	4.01	4.02
H6'	3.76	3.78	3.77	3.66	3.75	3.75	3.80	3.58	n.r	3.79	n.r
Lys Cϵ	50.9	50.9	50.9	50.9	50.9	50.9	50.9	n.r	n.r	50.9	n.r
Lys Hϵ	3.06	3.06	3.04	3.04	3.07	3.07	3.09	n.r	n.r	n.r	n.r

^a^ The two columns represent the two detected spin systems

^b^ Values of compound 5: Nα-(1-deoxy-d-fructos-1-yl)-l-alanine. For comparison with our data referenced to DSS, we added + 2.5 ppm to the values of Kaufmann referenced to TMS

^c^ Values of the peptide H-Lys([^13^C_6_]Fru)-Ala-Ala-Phe-OH

^d^ Values of compound 6 Nϵ-(1-deoxy-d-fructos-1-yl)-Nα-formyl-l-lysine. For comparison with our data, which is referenced to DSS, we added + 1.8 ppm to the values of Mossine, which were referenced to 1.4 dioxane

^e^ Values of d-fructosamine hydrochloride

^f^ Protons of the CH_2_ group (C1-H1) exchanged with deuterium of the solvent, leading to a mixture of CH_2_, CDH and CD_2_, of which the first two appeared with opposite sign in the multiplicity-edited spectrum plus the deuterium affected the ^13^C chemical shift, making it difficult to extract the exact peak position

n.r. not reported, n.d. not detected

There were few additional unassigned signals left with low intensity, whose ^13^C chemical shifts fit to previously reported values of the α-pyranose form. Unfortunately, the lack of ^1^H assignments for the α-pyranose form of fructoselysine [26] and glycated alanine [27] hampered identification. However, both ^1^H and ^13^C chemical shift assignments of d-fructoseamine hydrochloride were reported for all four cyclic forms, including the α-pyranose form [45]. These chemical shifts match our observed fourth set of signals, confirming their assignment to the α-pyranose form (Table S1). The abundances of the open forms were too low to be detectable in our spectra. Previous publications reported an abundance of < 1% [44].

The ^13^C chemical shift axis was crucial for separating and assigning the different species. In contrast, ^1^H-^1^H correlations like in the TOCSY spectrum do not allow the separation of the different species due to chemical shift degeneracies. Most signals overlap and are close to the diagonal.

**Fig. 4:**
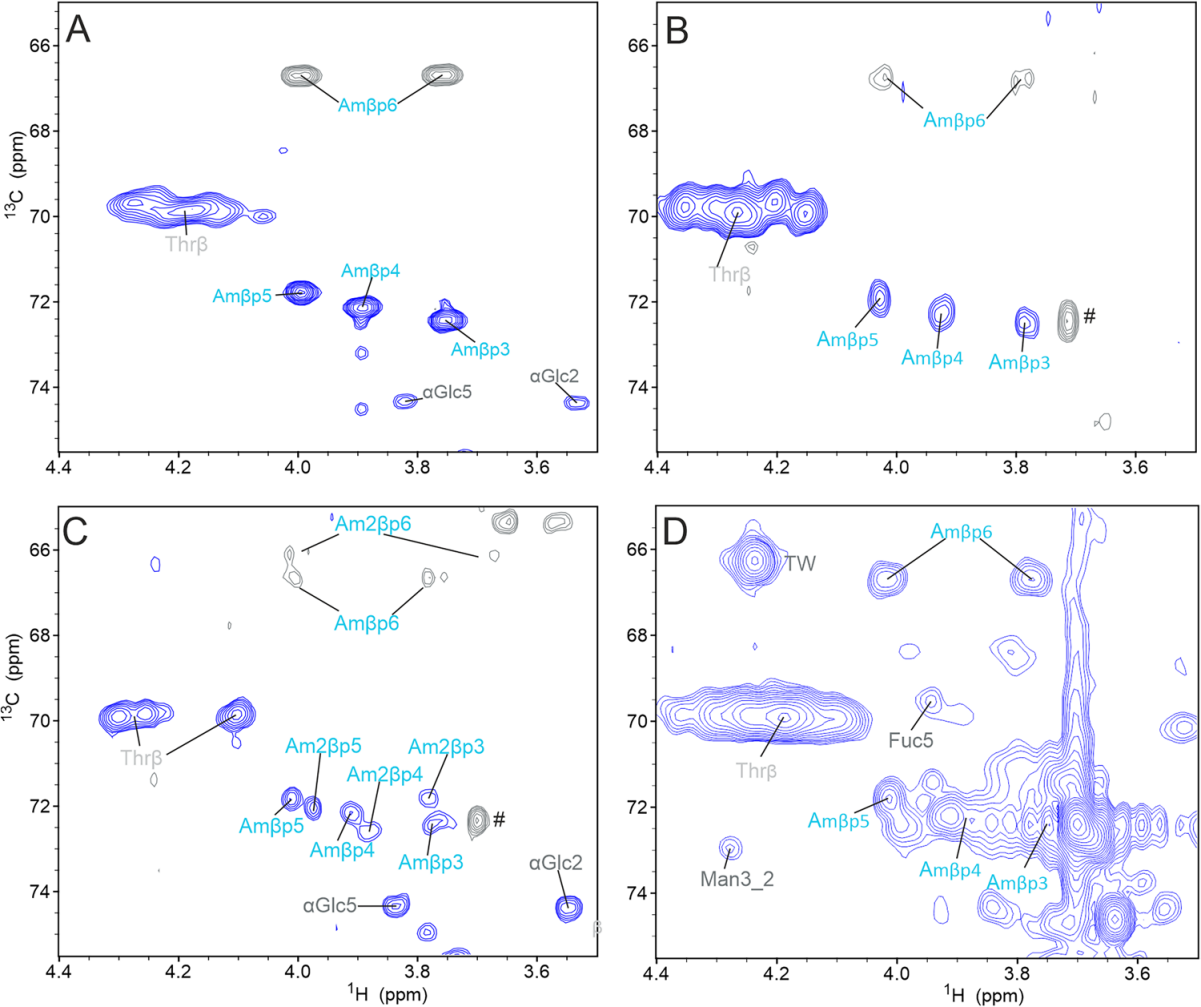
Glycation of model proteins monitored by ^1^H-^13^C HSQC spectra. (**A**) For comparison the region of interest of a ^1^H-^13^C HSQC spectrum of glycated BSA at a concentration of 2 mM measured with 128 transients, a recycle delay of 1 s and 2024 × 512 points. The HSQC was recorded with multiplicity-editing, signals of CH were positive (blue) and signals of CH_2_ negative (grey). The signals of the major β-pyranose form of the Amadori-product are labeled in cyan. For more details see Fig. 3. The cyan markers (Amβ) indicate the β-pyranose form of the Amadori product. (**B**) Comparable region of a ^1^H-^13^C HSQC spectrum of glycated lysozyme at a concentration of 3.85 mM measured with 400 transients, a recycle delay of 1 s and 2024 × 358 points. A small signal of polyethylenglycol (PEG) is indicated by #. (**C**) ^1^H-^13^C HSQC spectrum of glycated aprotinin at a concentration of 0.9 mM recorded with 400 transients, a recycle delay of 1 s and 2024 × 512 points. Here two sets of signals of signals corresponding to the β-pyranose form of the Amadori product are indicated one with Amβp and the other with Am2βp. A small signal of polyethylenglycol (PEG) is indicated by #. (**D**) ^1^H-^13^C HSQC spectrum of glycated rituximab at a concentration of 0.2 mM. The HSQC was recorded without multiplicity editing, with 140 transitions, 1024 × 256 points and a recycle delay of 2 s at 600 MHz. In addition to signals of the Amadori-product (cyan) cross-peaks of N-glycans are visible as well, but only two isolated glycan signals are labeled for simplicity. *TW* polysorbate

After detecting the Amadori-products in BSA, we wondered if the observed chemical shift correlations were characteristic and independent of the glycated protein. Therefore, we glycated lysozyme, aprotinin and the therapeutic monoclonal antibody rituximab under similar conditions at 40°C in the presence of phosphate (pH 6) and measured ^1^H-^13^C HSQC spectra under denaturing conditions. The therapeutic mAb rituximab was chosen to see if the signals of the Amadori product could be detected independently of glycosylation signals. We found the characteristic signals of the dominating β pyranose form in the ^1^H-^13^C HSQC spectrum of all glycated proteins (Fig. 4). Especially chemical shift correlations of C5-H5 at 71.8 /4.01 ppm and C4-H4 at 72.2 /3.89 ppm are visible with good intensity in all three proteins. The C6-H6 and C6-H6’ signals with the chemical shift pairs of 66.7 /4.01 ppm and 66.7 /3.76 ppm confirm the presence of the β pyranose form of fructoselysine, although their intensities are smaller than the C5-H5 and C4-H4 correlations. In particular, the chemical shift of C6 of the β-pyranose form seems to be unique and suitable as a specific marker for the Amadori product in denatured proteins. Lysozyme, with its four known glycation sites, shows very clearly all signals of the β-pyranose form at the same positions as in the BSA spectrum (Fig. 4A and B). Signals of the other forms were not visible. Surprisingly, glycated aprotinin (Fig. 4C) showed two sets of signals, which are both very similar to the signals of the β-pyranose form in BSA (Table I). Aprotinin is a rather small protein with only four lysine residues (Fig. S1), which has not been shown to react with glucose so far. However, glycation of aprotinin with lactose was reported and the results suggested that Lys15, which is crucial for its inhibitory function, is one of the glycation sites [46]. To elucidate the molecular basis for the two sets of signals of the β-pyranose form of the Amadori product in aprotinin, we applied intact and bottom-up HPLC–MS analysis (Figure S1 and Table S2). The analysis of the intact mass revealed one glycation as major species, but also significant amounts of two and three glycations per protein molecule together with an unglycated species (Fig. S1B). Bottom-up HPLC–MS analysis using legumain as protease [47] revealed also glycation at the N-terminus (Fig. S1C, D). As the first set of chemical shifts was most similar to the ones observed in the other glycated proteins, we assigned it to the glycation of Lys. The second set of signals is likely resulting from glycation of the N-terminus.

Because therapeutic mAbs can contain quite different populations of N-glycans with different terminal saccharides, we analyzed in addition the therapeutic mAb adalimumab. Figure S2 shows the region of interest in ^1^H-^13^C HSQC spectra of adalimumab before and after forced glycation. Strong signals of the main Amadori product appeared. Most importantly, characteristic signals of the main Amadori product did not overlap with signals of glycosylation, neither in rituximab, nor in adalimumab.

By integrating the volumes of isolated cross-peaks in glycated proteins and comparing the values of the Amadori-product with isolated protein signals, we calculated the percentage of glycation per molecule (Tables S3-S5). For glycated BSA, we compared the volume of the β-pyranose signals with the Cα-Hα signal of a serine that is followed by a proline at a characteristic position of 56.6 / 4.71 ppm [18]. The volumes of the isolated β-pyranose signals were 7–9 times higher than the volume of a normalized CH or CH_2_ group, which means that approximately 7–9 lysines per molecule are glycated. An earlier study based on chromatography coupled to MS found that 14 out of 59 lysine residues were susceptible to glycation [43]. In the case of glycated aprotinin, the volumes of both β-pyranose forms were compared to the volume of the Cα-Hα signal of the four phenylalanines in the sequence, resulting in an average glycation of ~ 22% of the molecules (both species combined). For glycated lysozyme, comparing the isolated Amadori signals to the volume of an isolated Cα-Hα signal revealed that on average 33% of the proteins were glycated. In glycated rituximab, only the C6-H6 signals of the β-pyranose form were isolated and could be integrated. Comparing the C6-H6 signal volumes to the volume of the Cγ-Hγ of glutamic acid revealed that on average 4 lysines were glycated per mAb.

### Glycation in Natural Proteins

**Fig. 5:**
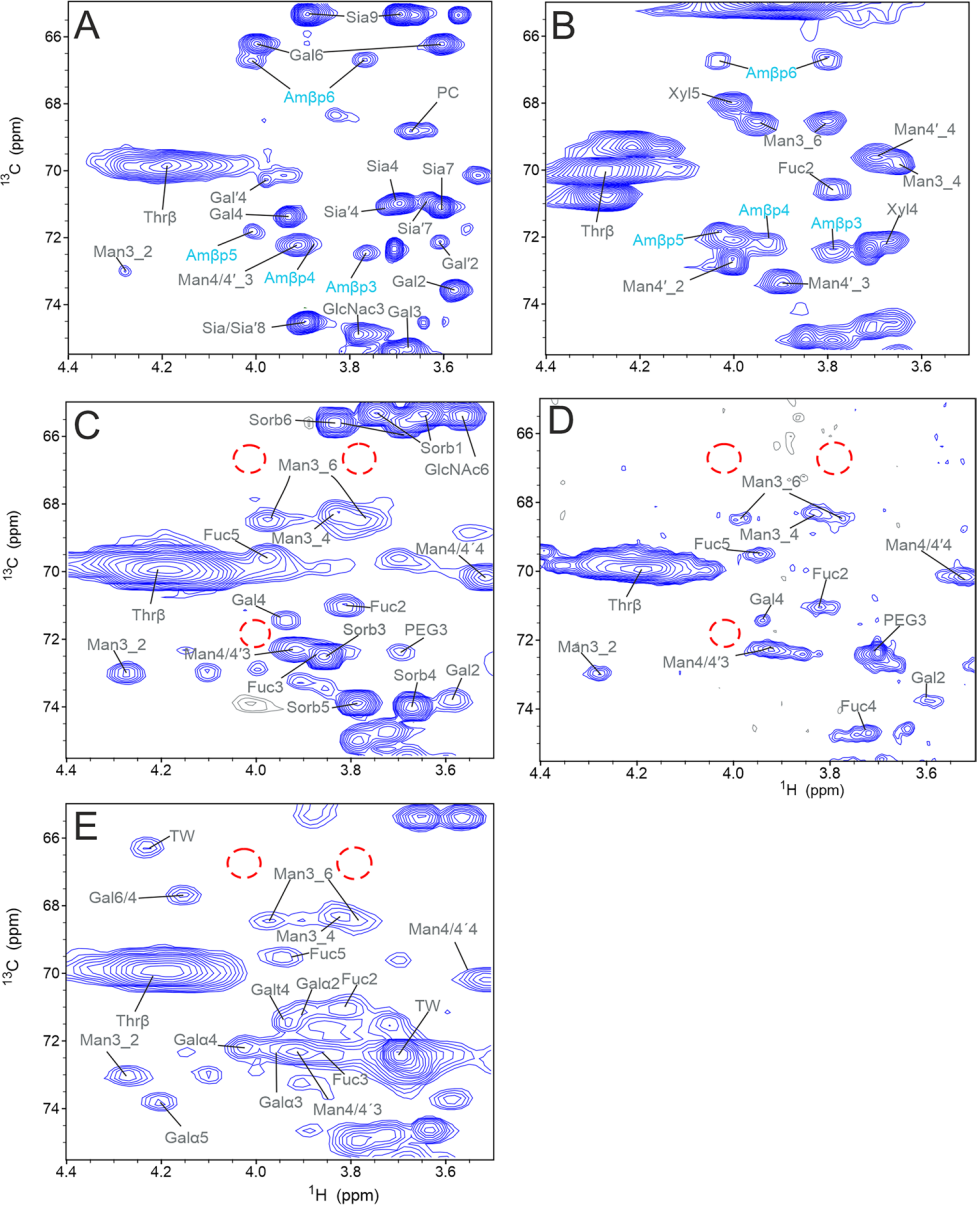
Investigation of glycation in natural and therapeutic proteins by 2D ^1^H-^13^C HSQC spectra. (**A**) ^1^H-^13^C HSQC spectra of human serum albumin, at a concentration of 2.5 mM, showing typical correlations of the β-pyranose form of the Amadori-product (cyan label). The spectrum was measured with 112 transients, 2048 × 840 points and a recycle delay of 1 s at 900 MHz. Grey labels indicate the assignments of glycan signals. PC = phosphocholine, Sia and Gal are part of a Neu5Acα2,6Galβ1,4Man antenna and Sia′ and Gal′ are part of a Neu5Acα2,3Galβ1,4Man antenna. (**B**) ^1^H-^13^C HSQC spectrum of bromelain, at a concentration of 3.5 mM, recorded with 192 transients, 2048 × 700 points and a recycle delay of 1 s at 500 MHz. The natural glycation signals of the β-pyranose form are labeled cyan. (**C**) Comparable region of a ^1^H-^13^C HSQC spectrum of denosumab recorded with 160 transients, 1024 × 256 points and a recycle delay of 2 s at 700 MHz. The red dotted circles mark the characteristic positions of the β-pyranose form of the Amadori product. Sorb = sorbitol (**D**) ^1^H-^13^C HSQC spectrum of trastuzumab measured with 128 transients, 2048 × 800 points and a recycle delay of 2 s at 600 MHz. (**E**) ^1^H-^13^C HSQC spectrum of cetuximab at a concentration of 40 mg/mL, recorded with 360 transients, 2048 × 512 points and a recycle delay at 1.3 s at 500 MHz.

Beside artificially glycated proteins, we also investigated natural proteins and therapeutic mAbs without artificial treatment. For this purpose, we studied human serum albumin, bromelain, and the biotherapeutics denosumab, cetuximab and trastuzumab using ^1^H-^13^C HSQC spectra under denaturing conditions (Fig. 5). Commercial human serum albumin (isolated from serum) and the protease bromelain (isolated from pineapple) showed ^1^H-^13^C correlations that match the major β-pyranose form of the Amadori-product (Fig. 5A-B). The human serum albumin likely contained impurities of other serum proteins, as was shown earlier [48]. This is supported by intense N-glycosylation signals although the protein sequence lacks an N-glycosylation sequon (Fig. 5A). None of the therapeutic mAbs showed any signs of the Amadori product (Fig. 5C-E), even for a mAb that was reported to be glycated [38]. Despite the high complexity of the ^1^H-^13^C HSQC spectrum and many correlations of the containing glycans, the typical regions for C5-H5 (71.8/4.01 ppm), C6-H6 (66.7 /4.01 ppm) and C6-H6′ (66.7 /3.76 ppm) were empty. Denosumab was described to be glycated [38], but from our NMR data (see Fig. 5C), we concluded that either the glycation level was below the detection limit, or another form of modification was present, different from glycation with glucose.

For quantification, we integrated the signals of isolated cross-peaks of the β-pyranose form of the Amadori-product and compared them to volumes of isolated amino acid random-coil signals. For HSA, comparing integrals of the Amadori product with normalized CH and CH_2_ reference signals revealed that on average 1.5 lysines were glycated per molecule. A similar analysis for bromelain showed that an average 42% of the protein molecules are glycated. This investigation also revealed that some proteins showed signals that partially superimposed with the C3-H3 and C4-H4 signals of the major Amadori product. However, the C5-H5, C6-H6 and C6-H6′ correlations did not superimpose with other signals, and are therefore the best indicators for glycation with glucose.

## Discussion

MS coupled to liquid chromatography is so far the standard method for high-throughput detection of PTMs, but lacks specificity, since it is hard to distinguish between two isobaric modifications due to the same mass. In contrast, NMR spectroscopy and more specifically chemical shift patterns in 2D spectra measured under denaturing conditions, enable the unambiguous identification of many PTMs [7–12, 19]. Here we demonstrated that glycation products in model proteins show unique chemical shift correlations that are not superimposed by other protein or glycan signals. The chemical shift correlations of C5-H5, C6-H6 and C6-H6′ of the β-pyranose form of the Amadori-product are suited to unambiguously detect glycation. These signals are found consistently at the same place and are not overlapping with other signals. Comparison with spectra of other mAbs and Fc-fusion proteins covering a large variety of different N- and some O-glycan populations showed that these signals are not impaired by glycan signals.

In previous investigations, we estimated the detection limit for a PTM observed by ^1^H-^13^C HSQC spectra for 5 mm NMR tubes to be approx. 55 µmol/L, which corresponds for a 500 μL sample to an absolute amount of 28 nmol [9]. For a mAb with a concentration of 220 µmol/L, which is close to saturation, this corresponds to a limit of detection of 25% of modification per mAb (two heavy chains and two light chains) [9]. The limit is generally independent of the kind of modification, but dependent on the concentration of the protein, the sensitivity of the spectrometer and the time of measurement. The typical measurement times to reach the estimated limit were 1 day at a 900 MHz spectrometer with a cryogenic probe or 2 days at a 600 MHz spectrometer with a cryogenic probe. Studies of isolated Fc and Fab domains of mAbs estimated a detection limit of 10% using a protein concentration of ca. 660 µmol/L, which resulted in a lowest detectable concentration of 66 µmol/L [19], very similar to our previous estimation. This detection limit was obtained by measuring 11 h at an 850 MHz spectrometer with cryogenic probe. The higher the concentration of the investigated protein, the lower the percentage of modification per molecule that can be detected. With a concentration of 0.9 mM (like aprotinin in our case) in principle, modifications with an abundance above 6% can be detected. With a concentration of 3.85 mM, as in our lysozyme sample, an abundance of as low as 0.7% can still be detected.

Denosumab was earlier reported to be glycated [38], but in our case the spectra lacked any signs of glycation, suggesting a glycation level below our NMR detection limit, or a modification with another reducing sugar other than glucose. However, it is also possible, that the glycation was lost during the sample preparation due to its reversibility [28]. Due to our sample preparation being much shorter than the previously reported half-life of Amadori products [28], this is unlikely.

Compared to already established methods, the strength of our method is the specificity. We are able to clearly determine Amadori products if the concentration of the modification is above 55 µmol/L. We are able to distinguish between an added hexose and a glycation with an isobaric weight of + 162 Da. The sample preparation is straightforward and depending on the concentration of the protein, the measurement takes 1–2 days on a spectrometer with cryo probe. The results that we obtained were comparable in all investigated denatured proteins.

## Conclusion

This work presents an extensive NMR characterization of glucose-induced glycation in model proteins studied under denaturing conditions. Complete ^1^H and ^13^C chemical shift assignments of the four major Amadori products are provided. The most abundant form (70%) gives characteristic ^1^H-^13^C correlations in ^1^H-^13^C HSQC spectra, which are suited for a fast and unambiguous identification of glycation in protein therapeutics by 2D NMR spectroscopy. This straightforward 2D NMR approach works also for very large proteins like therapeutic mAbs. Due to its complementarity to MS-based approaches, it is suitable as a gold standard for cross-validation studies.

## Supplementary Information

Below is the link to the electronic supplementary material.

Supplementary file1 (PDF 791 KB)

## Data Availability

All data generated or analysed during this study are included in this published article (and its supplementary information files).
