# Supplementary material for: Unambiguous Identification of Glucose-Induced Glycation in mAbs and other Proteins by NMR Spectroscopy
Source: Pharm Res. 2022 Dec 13;40(6):1341–53. doi: 10.1007/s11095-022-03454-0 (PMC10338404; doi:10.1007/s11095-022-03454-0)
Supplement: Supplementary file 1 — Supplementary file1 (PDF 791 KB) [file 11095_2022_3454_MOESM1_ESM.pdf]

Supplemental Material

Unambiguous identification of glucose-induced glycation in mAbs and other proteins by NMR spectroscopy

Jennifer E. Moises, Christof Regl, Arthur Hinterholzer, Christian G. Huber, and Mario Schubert

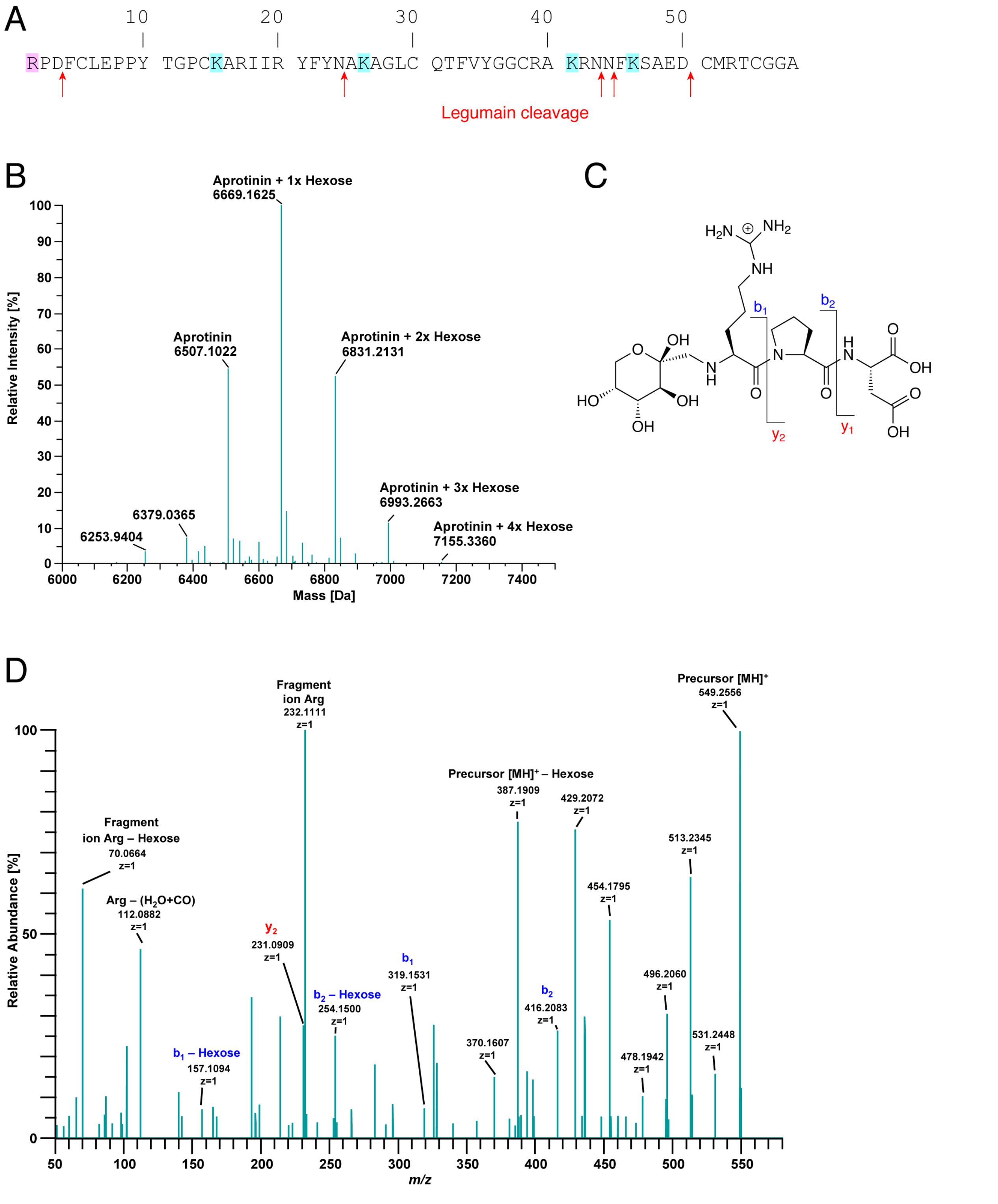

Figure S1. Annotation of glycated aprotinin isoforms by HPLC-MS analysis after 1 week incubation at 40°C with 500 mmol·L^{-1} D-glucose. A) Sequence of aprotinin with potential glycation sites indicated. The four containing Lys residues are color-coded in cyan, the N-terminus in pink. In addition cleavage sites of the protease legumain are indicated by red arrows. **B)** Annotation of aprotinin isoforms in a deconvoluted mass spectrum of intact aprotinin by HPLC-MS analysis. **C)** Schematic presentation of the glycated N-terminal peptide with some characteristic fragmentations. D) The glycated N-terminal peptide

was fragmented employing higher-energy collisional induced dissociation, resulting in fragmentation of the peptide backbone (b- and y-ions) as well as in the Amadori-product (–Hexose).

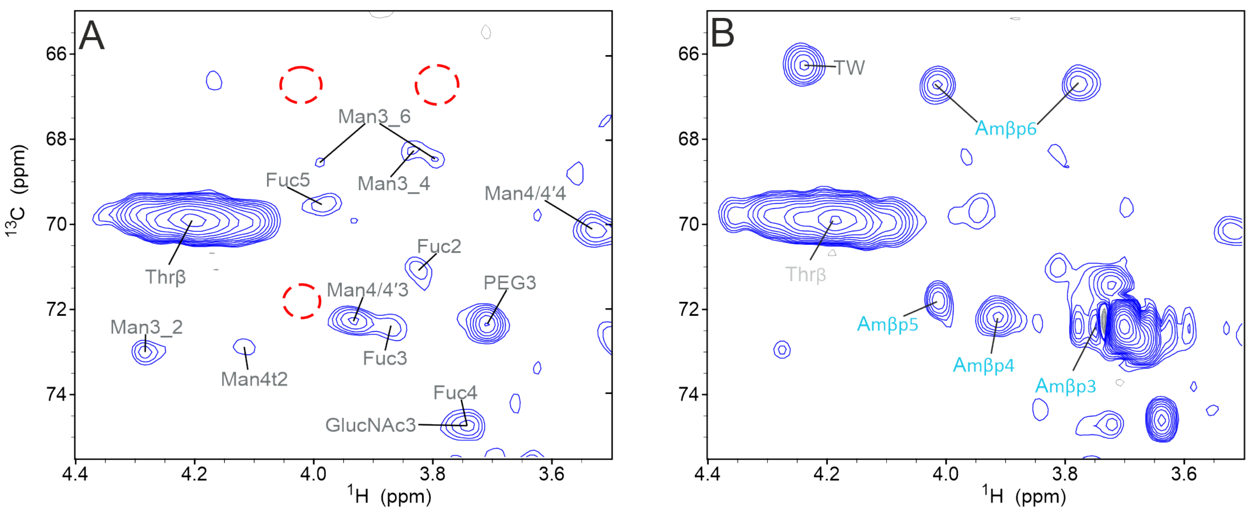

Figure S2. Investigation of glycation of the therapeutic mAb adalimumab by 2D ^{1}H-^{13}C HSQC spectra. A) ^{1}H-^{13}C HSQC spectra of the untreated adalimumab, at a concentration of 0.22 mM, showing typical signals of glycosylation. The regions of most characteristic glycation signals indicated by red dotted circles are empty. The spectrum was recorded with 140 transients, a recycle delay of 2 sec and 1024 × 256 points. **B)** ^{1}H-^{13}C HSQC spectra of the glycated adalimumab, measured with 140 transients, a recycle delay of 2 sec and 1024 × 256 points..

Table S1. Experimental chemical shifts of the two furanose forms and the two pyranose forms of the Amadori-product, observed in glycated model proteins in comparison to previously published data (1-4).

	BSA	Kaufmann 2016 [a]	Kapczynska 2011[b]	Mossine 1994 [c]	Mossine 2009 [d]
**α-furanose**					
**C1**	n.d	52.5	55.1	53.5	46.2
**C2**	104.6	104.5	n.r	104.5	104.7
**C3**	85.1	85.1	83.4	85.2	85.1
**C4**	78.7	78.7	78.4	78.7	78.9
**C5**	85.3	85.3	85	85.0	85.1
**C6**	63.5	63.6	63.4	63.4	63.7
**H1**	n.d	3.15	n.r	n.r	3.26
**H1'**	n.d	3.12	n.r	n.r	3.26
**H3**	4.20	4.02	n.r	n.r	4.22
**H4**	4.02	3.84	n.r	n.r	4.02
**H5**	4.11	3.94	n.r	n.r	4.12
**H6**	3.82	3.66	n.r	n.r	3.84
**H6'**	3.69	3.53	n.r	n.r	3.71
**β-furanose**					
**C1**	n.d	53.6	53.7	54.9	47.3
**C2**	101.6	101.5	n.r	101.5	101.7
**C3**	80.7	80.6	83.5	80.5	80.4
**C4**	76.9	76.8	76.6	76.8	77.1
**C5**	83.8	83.6	84.9	83.5	83.6
**C6**	64.6	64.6	64.4	64.5	64.7
**H1**	n.d	3.15	n.r	n.r	3.24
**H1'**	n.d	3.10	n.r	n.r	3.24
**H3**	4.02	3.86	n.r	n.r	4.05
**H4**	4.11	3.92	n.r	n.r	4.13
**H5**	3.88	3.70	n.r	n.r	3.89
**H6**	3.79	3.62	n.r	n.r	3.82
**H6'**	3.68	3.49	n.r	n.r	3.69
**α-pyranose**					
**C1**	n.d	49.9	n.r	51,6	43.6
**C2**	98.9	99.0	n.r.	98.7	98.8
**C3**	73.2	73.0	n.r.	74.3	73.1
**C4**	74.5	74.8	n.r.	73.0	74.5
**C5**	68.5	68.7	n.r.	68.1	68.5
**C6**	65.4	65.8	n.r.	65.2	65.5
**H1**	n.d	3.18	n.r	n.r	3.36
**H1'**	n.d	3.13	n.r	n.r	3.31
**H3**	3.89	n.r.	n.r.	n.r.	3.91
**H4**	3.89	n.r.	n.r.	n.r.	3.90
**H5**	4.03	n.r.	n.r.	n.r.	4.04

**H6**	3.89	n.r.	n.r.	n.r.	3.89
**H6'**	3.69	n.r.	n.r.	n.r.	3.74
**β-pyranose**					
**C1**	55.6	54.5	55.6	55.5	48.0
**C2**	98.2	98.0	n.r	98.1	98.1
**C3**	72.4	72.6	72.2	72.3	72.4
**C4**	72.1	72.0	72.1	72.0	72.1
**C5**	71.8	71.6	71.5	71.6	71.7
**C6**	66.7	66.6	66.6	66.6	66.7
**H1**	3.29	3.14	n.r	n.r	3.28
**H1'**	n.d	3.11	n.r	n.r	3.24
**H3**	3.75	3.57	n.r	n.r	3.75
**H4**	3.89	3.71	n.r	n.r	3.91
**H5**	4.00	3.82	n.r	n.r	4.03
**H6**	3.99	3.84	n.r	n.r	4.02
**H6'**	3.76	3.58	n.r	n.r	3.78

[a] values of compound 5: N^{α}-(1-deoxy-D-fructos-1-yl)-L-alanine, for comparison with our data (referenced to DSS) we added +2.5ppm to the values of Kaufmann referenced to TMS
[b] values of the peptide H-Lys([$^{13}C_6$]Fru)-Ala-Ala-Phe-OH
[c] values of compound 6 N^{ϵ}-(1-deoxy- D-fructos-1-yl)-N^{α}-formyl-L-lysine. For comparison with our data referenced to DSS, we added +1.8ppm to the values of Mossine 1994, which were referenced to 1.4 dioxane
[d] values of D-fructosamine hydrochloride
n.r not reported
n.d not detected

Table S2: Annotation of aprotinin isoforms in a deconvoluted mass spectra of intact aprotinin by HPLC-MS analysis after 1 week stressing at 40°C with 500 mmol·L^{-1} D-glucose.

Monoisotopic Mass [Da]	Sum Intensity	Relative Abundance [%]	Fractional Abundance [%]	Annotation	Theoretical mass [Da]	Δppm
6669.1625	172696428255.2	100.00	32.82	Aprotinin + 1x Hexose	6669.0924	10.51
6507.1022	93949992967.0	54.40	17.86	Aprotinin	6507.0414	9.35
6831.2131	90401809577.2	52.35	17.18	Aprotinin + 2x Hexose	6831.1471	9.66
6685.1503	25544755429.3	14.79	4.86	Aprotinin + 1x Hexose + 1x Oxidation	6685.0891	9.15
6993.2663	19495256402.9	11.29	3.71	Aprotinin + 3x Hexose	6993.1999	9.50
6379.0365	12755863935.9	7.39	2.42	Aprotinin - Gly-Ala (C-term)	6378.9828	8.42
6847.2075	12385226032.3	7.17	2.35	Aprotinin + 2x Hexose + 1x Oxidation	6847.1420	9.57
6523.0977	12225709620.2	7.08	2.32	Aprotinin + 1x Oxidation	6523.0363	9.41
6541.0974	10775220491.7	6.24	2.05	Aprotinin- Gly-Ala (C-term) + 1x Hexose	6541.0357	9.44
6598.1109	10709377190.8	6.20	2.04	Aprotinin- Ala (C-term) + 1x Hexose	6598.0571	8.15
6732.1560	9999571824.5	5.79	1.90	-	-	-
6436.0458	8624314019.3	4.99	1.64	Aprotinin - Ala (C-term)	6436.0043	6.45
6416.0056	6017697473.6	3.48	1.14	Aprotinin- Arg-Pro (N-term) + 1x Hexose	6415.9404	10.17
6253.9404	5711987647.1	3.31	1.09	Aprotinin - Arg-Pro (N-term)	6253.8875	8.45
6894.2119	5033640037.9	2.91	0.96	-	-	-
6760.1734	4186437039.6	2.42	0.80	Aprotinin - Ala (C-term) + 2x Hexose	6760.1099	9.38
6703.1629	3676918846.3	2.13	0.70	Aprotinin - Gly-Ala (C-term) + 2x Hexose	6703.0885	11.11
6570.0954	3350788190.9	1.94	0.64	-	-	-
6653.1488	3150805619.7	1.82	0.60	-	-	-
6814.2078	2577099206.9	1.49	0.49	-	-	-
6614.1191	2248264300.6	1.30	0.43	Aprotinin - Ala (C-term) + 1x Hexose + 1x Oxidation	6614.0520	10.15
6578.0955	1979094471.2	1.15	0.38	Aprotinin - Arg-Pro (N-term) + 2x Hexose	6577.9932	15.55
6395.0436	1829485819.4	1.06	0.35	Aprotinin - Gly-Ala (C-term) + 1x Oxidation	6394.9777	10.29
6711.1793	1353718361.1	0.78	0.26	Aprotinin + 1x Hexose + 1x Acetylation	6711.1048	11.10
6557.1033	1271475177.6	0.74	0.24	Aprotinin- Gly-Ala (C-term) + 1x Hexose + 1x Oxidation	6557.0306	11.09
7009.2725	1132381559.0	0.66	0.22	Aprotinin + 3x Hexose + 1x Oxidation	7009.1948	11.09
6626.1411	1119412682.1	0.65	0.21	-	-	-
6452.0585	941385845.5	0.55	0.18	Aprotinin - Ala (C-term) + 1x Oxidation	6451.9992	9.19
6491.0814	380246748.0	0.22	0.07	-	-	-
6705.1325	301972329.0	0.17	0.06	-	-	-
7155.3360	297838445.4	0.17	0.06	Aprotinin + 4x Hexose	7155.2527	11.64

Table S3. Reference Signals for the quantification of glycation.

Protein	Multiplicity	Reference signal for integration	$^{13}C/^{1}H$ resonances (ppm)	Volume	Number of residues in sequence	Normalized volume per proton
**BSA**	CH	Ser 109 Cα-Hα[a]	56.6/4.71	$1.18\ 10^{8}$	1	$1.18\ 10^{8}$
	CH_2	Arg Cδ-Hδ	27.4/1.65	$-4.38\ 10^{8}$	23	$9.53\ 10^{7}$
**Lysozym**	CH	Thr 69 Cα-Hα[a]	60.1/4.60	$3.93\ 10^{8}$	1	$3.93\ 10^{8}$
**Aprotinin**	CH	Phe Cα-Hα	57.6/4.63	$7.81\ 10^{10}$	4	$1.95\ 10^{10}$
**Rituximab**	CH_2	Glu Cγ-Hγ	36.4/2.30	$-2.78\ 10^{9}$	62[b]	$-4\text{-}48\ 10^{7}$
**HSA**	CH	Ile Cβ-Hβ	38.9/1.90	$3.89\ 10^{8}$	9	$4.33\ 10^{7}$
	CH_2	Gly Cα-Hα[a]	45.2/3.97	$2.20\ 10^{9}$	13	$8.46\ 10^{7}$
**Bromalein**	CH	Ile Cβ-Hβ	39.1/1.88	$1{,}94\ 10^{10}$	20	$9{,}70\ 10^{8}$
	CH_2	Gly Cα-Hα	45.2/4.01	$6.62\ 10^{9}$	28	$1.18\ 10^{8}$

[a] these residues are followed by a proline and all found at a characteristic region after Wishart et al 1995 (5)
[b] within the sequences of two heavy chains and two light chains

Table S4. Integrated volumes of the β-pyranose form of the Amadori product

	BSA	Aprotinin set 1	Aprotinin set 2	Lysozyme	Rituximab	HSA	Bromelain
**C3-H3**	1.10E+09	n.i.	3.44E+09	1.37E+08	n.i.	9.56E+08	1.33E+08
**C4-H4**	1.08E+09	4.69E+09	5.44E+09	1.37E+08	n.i.	3.07E+08	n.i.
**C5-H5**	1.01E+09	4.86E+09	4.29E+09	1.18E+08	n.i.	7.51E+07	n.i.
**C6-H6**	-6.93E+08	n.i.	n.i.	n.i.	-8.91E+07	1.07E+08	1.11E+08
**C6-H6'**	-7.37E+08	n.i.	n.i.	n.i.	-9.23E+07	1.06E+08	1.34E+08

n.i. not integrable

Table S5. Percentage of glycation in a molecule calculated with the values of Table S3 and S4

	BSA	Lysozyme	Aprotinin set 1	Aprotinin set 2	Rituximab	HSA	Bromelain
**C3-H3**	9.32	0.35	n.i.	0.17	n.i	1.66	0.51
**C4-H4**	9.15	0.35	0.23	0.27	n.i	n.i	n.i
**C5-H5**	8.56	0.30	0.24	0.21	n.i	1.82	n.i
**C6-H6**	7.27	n.i	n.i	n.i	3.97	1.26	0.34
**C6-H6'**	7.73	n.i	n.i	n.i	4.12	1.25	0.41
**On average**	8.41	0.33	0.23	0.21	4.05	1.50	0.42

n.i. not integrable

Supplementary References:

1. Mossine VV, Glinsky GV, Feather MS. The preparation and characterization of some Amadori compounds (1-amino-1-deoxy-D-fructose derivatives) derived from a series of aliphatic omega-amino acids. Carbohydr Res. 1994;262(2):257-70.
2. Kaufmann M, Meissner PM, Pelke D, Mügge C, Kroh LW. Structure–reactivity relationship of Amadori rearrangement products compared to related ketoses. Carbohydr Res. 2016;428:87-99.
3. Kapczyńska K, Stefanowicz P, Jaremko L, Jaremko M, Kluczyk A, Szewczuk Z. The efficient synthesis of isotopically labeled peptide-derived Amadori products and their characterization. Amino Acids. 2011;40(3):923-32.
4. Mossine VV, Barnes CL, Mawhinney TP. Structure of D-fructosamine hydrochloride and D-fructosamine hydroacetate. J Carbohydr Chem. 2009;28(5):245-63.
5. Wishart DS, Bigam CG, Holm A, Hodges RS, Sykes BD. 1H, 13C and 15N random coil NMR chemical shifts of the common amino acids. I. Investigations of nearest-neighbor effects. J Biomol NMR. 1995;5(1):67-81.
